# Targeting EGF-receptor(s) - STAT1 axis attenuates tumor growth and metastasis through downregulation of MUC4 mucin in human pancreatic cancer

**DOI:** 10.18632/oncotarget.3286

**Published:** 2014-12-31

**Authors:** Parthasarathy Seshacharyulu, Moorthy P. Ponnusamy, Satyanarayana Rachagani, Imayavaramban Lakshmanan, Dhanya Haridas, Ying Yan, Apar K. Ganti, Surinder K. Batra

**Affiliations:** ^1^ Department of Biochemistry and Molecular Biology, University of Nebraska Medical Center, Omaha, NE, USA; ^2^ Fred and Pamela Buffett Cancer Center, Eppley Institute for Research in Cancer and Allied Diseases, University of Nebraska Medical Center, Omaha, NE, USA; ^3^ Department of Radiation Oncology, University of Nebraska Medical Center, Omaha, NE, USA; ^4^ Department of Internal Medicine, VA Nebraska-Western Iowa Health Care System and University of Nebraska Medical Center, Omaha, NE, USA

**Keywords:** Afatinib, Canertinib, Pancreatic cancer, MUC4, EGFR

## Abstract

Transmembrane proteins MUC4, EGFR and HER2 are shown to be critical in invasion and metastasis of pancreatic cancer. Besides, we and others have demonstrated *de novo* expression of MUC4 in ~70-90% of pancreatic cancer patients and its stabilizing effects on HER2 downstream signaling in pancreatic cancer. Here, we found that use of canertinib or afatinib resulted in reduction of MUC4 and abrogation of *in vitro* and *in vivo* oncogenic functions of MUC4 in pancreatic cancer cells. Notably, silencing of EGFR family member in pancreatic cancer cells decreased MUC4 expression through reduced phospho-STAT1. Furthermore, canertinib and afatinib treatment also inhibited proliferation, migration and survival of pancreatic cancer cells by attenuation of signaling events including pERK1/2 (T202/Y204), cyclin D1, cyclin A, pFAK (Y925) and pAKT (Ser473). Using *in vivo* bioluminescent imaging, we demonstrated that canertinib treatment significantly reduced tumor burden (*P*=0.0164) and metastasis to various organs. Further, reduced expression of MUC4 and EGFR family members were confirmed in xenografts. Our results for the first time demonstrated the targeting of EGFR family members along with MUC4 by using pan-EGFR inhibitors. In conclusion, our studies will enhance the translational acquaintance of pan-EGFR inhibitors for combinational therapies to combat against lethal pancreatic cancer.

## INTRODUCTION

Pancreatic cancer is the fourth leading cause of cancer associated mortality in the US with a very poor five-year survival of 6% [1]. The US FDA has approved erlotinib for the treatment of advanced pancreatic cancer in combination with gemcitabine [2]. Multiple clinical trials have been conducted with various anti-epidermal growth factor receptor (EGFR) monoclonal antibodies, anti-VEGF and farnesyl transferrase inhibitors, but with no evidence of improved efficacy [3]. Recently the US-FDA have approved nanoparticle albumin-bound (nab)-paclitaxel (Abraxane, Celgene), in combination with gemcitabine, for the first-line treatment of metastatic pancreatic cancer [4]. On the other hand, use of single agent that targets specific molecule in *in vivo* studies of pancreatic cancer has provided modest effects in specific genetic background. Thus combination studies providing multiple targeting effects are warranted to improve the pancreatic cancer patient survival.

Aberrant expression of cell surface mucins is a hallmark of epithelial cancers [5]. Among various mucins, MUC4, a high molecular weight membrane bound mucin, is one of the top most differentially overexpressed (4^th^ gene) in pancreatic cancer [6, 7]. Over a period of one decade, we and others have shown that MUC4 is undetectable in normal pancreas, while its expression increases progressively with the advancement of pancreatic cancer [8, 9]. We and others have also shown the differential overexpression of MUC4 in human primary pancreatic cancer tissues ranging from 70-90% [9, 10]. Furthermore, earlier studies from our group have shown that MUC4 enhances invasion and metastasis of pancreatic cancer [11, 12]. Similarly, EGFR family members such as HER1/EGFR (40-70%) and HER2 (22%) are overexpressed in pancreatic cancer and are associated with poor prognosis [13]. Our earlier studies have shown that MUC4, a transmembrane mucin, interacts, stabilizes and activates HER2 mediated downstream signaling in pancreatic and ovarian cancer cells [14, 15]. It has been proposed that MUC4 with its three EGF domain repeats may serve as ligand for HER2 [16]. On the other hand, we and others have also demonstrated the role of MUC4 in the mediation of gemcitabine resistance in pancreatic cancer [17, 18]. Moreover, MUC4 transcriptional upregulation was found to be activated by EGF mediated signaling response along with activation of intracellular tyrosine kinase in pancreatic cancer cells [19].

The concept of utilizing EGFR targeting small molecule tyrosine kinase inhibitors (TKIs) as a molecular therapeutic agent was first proposed by Mendelsohn *et al* [20]. However, several preclinical and clinical studies evaluating the therapeutic efficacy of drugs targeting EGFR such as erlotinib and gifatinib resulted in poor patient outcome. Additionally, observed benefits of HER2 targeted humanized monoclonal antibody, Herceptin, is also marginal and restricted to a subset of pancreatic cancer patients [21]. Thus, targeting one or more EGFR family members is an alternative approach to enhance patient's response to cancer therapy. There are two major classes of TKIs, reversible TKIs that binds to the active sites of EGFR kinase domain and irreversible TKIs that binds to cysteine residues in the ATP binding sites of kinase domains of all the EGFR family members (pan-EGFR inhibitors) [20].

Canertinib (CI 1033) is an irreversible TKI of all the EGFR family members. It not only inhibits tyrosine phosphorylation but also enhances ubiquitinylation and accelerates endocytosis [22]. Canertinib induces growth inhibition and apoptosis of melanoma, esophageal, breast and colon cancer cells [22-26]. Preclinical data shows that treatment of athymic nude mice bearing xenografts of various tumors with canertinib results in a significant suppression of tumor growth [23, 27]. Similarly, afatinib (BIBW2992) is another irreversible pan-EGFR inhibitor that has been shown to be effective in inhibiting the tumor growth of lung and breast cancer, both *in vitro* and *in vivo* [28-30].

In the present study, for the first time, we have evaluated the role of EGFR family pan-inhibitors canertinib and afatinib in the inhibition of MUC4-mediated invasion, motility and metastasis of pancreatic cancer cells. Our study provides a strong evidence of profound effects of irreversible pan-EGFR inhibitors (TKIs) in down regulating MUC4 mucin through its effect on the EGFR family proteins resulting in decreased pancreatic cancer cell proliferation, survival and migration. The *in vitro* studies were further corroborated with decreased tumorigenesis and metastasis related cell behavior in an *in vivo* orthotopic model of pancreatic cancer. Additionally, the MUC4 protein expression was not inhibited by erlotinib, a reversible EGFR inhibitor, in pancreatic cancer cells. Through this preclinical study, we provide evidences for the use of irreversible TKIs as a novel approach to reduce tumor burden as well as incidence of metastasis by down regulating MUC4 in advanced pancreatic cancer patients.

## RESULTS

### Canertinib and afatinib affects specifically EGFR and HER2 activities and expression in pancreatic cancer cells

**Figure 1:**
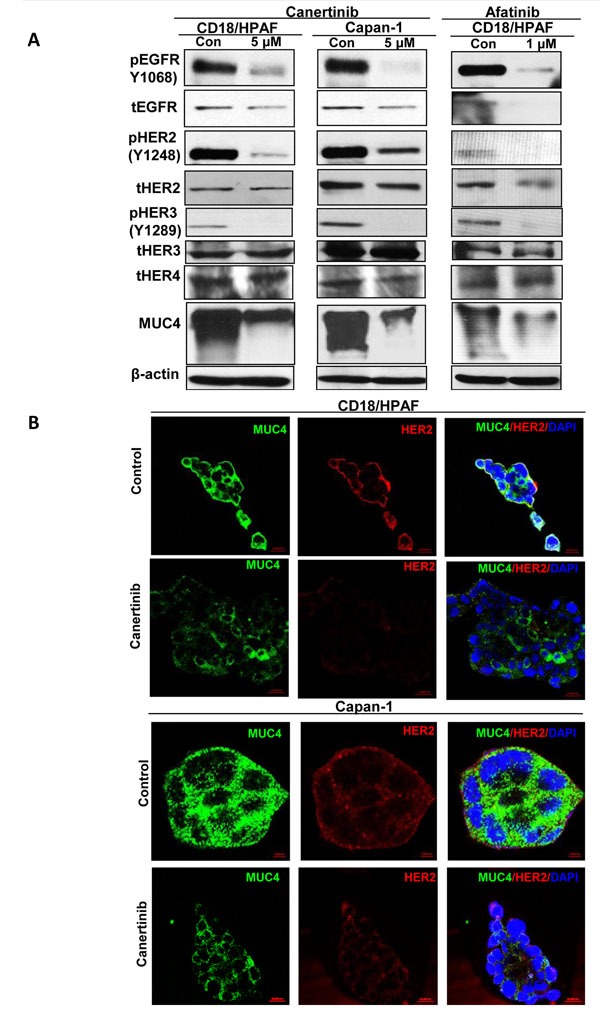
Inhibition of EGFR family members and MUC4 down regulation by pan EGFR inhibitors canertinib and afatinib in pancreatic cancer cells (A). CD18/HPAF and Capan-1 cells were treated with canertinib and afatinib (5 and 1 μM concentration) for 24 hours and control cells were treated with 0.01% DMSO in complete medium. The cell lysates (40μg) were separated by 10% SDS–PAGE and analyzed by Immunoblotting for phospho-EGFR (Y1068), phospho-HER2 (Y1248), phospho-HER3 (Y1289) and total forms of EGFR and HER2 antibodies at indicated concentrations. In parallel, protein lysates were resolved using 2% SDS agarose gel and MUC4 mucin protein was detected by western blot analysis employing anti-MUC4 (8G7) monoclonal antibody. Canertinib and afatinib decreases phosphorylated and total forms of EGFR and HER2, phosphorylated form of HER3, but no change in the expression pattern of blots probed with HER3 and HER4 specific antibodies, between the treatment and control lysates. Canertinib and afatinib significantly decreases MUC4 expression level in both the cell lysates as observed by western blots analysis. The membranes were re-probed with β-actin antibody for loading control. (B). Panel of immunofluorescence images of pancreatic cancer cell lines CD18/HPAF and Capan-1 grown on sterilized glass coverslips, methanol fixed and stained against MUC4 and HER2 specific antibodies; followed by fluorescently labeled secondary antibodies conjugated with FITC (MUC4), Texas red (HER2) and the cell nuclei were counterstained with DAPI. The images depict a representative of decreased membranous localization pattern of MUC4 and HER2 in canertinib treatment compared to control cells. Note that the change in the color from red to yellow as observed in the control cells are a representation of MUC4 interaction with HER2, which is absent in canertinib treated cells.

First, we performed a MTT assay to investigate the dose dependent inhibitory effect of canertinib, afatinib and erlotinib TKIs on pancreatic cancer cells (CD18/HPAF and Capan-1) for an incubation period of 24 h. The IC_50_ was approximately 5 μM concentration of canertinib in both CD18/HPAF and Capan-1 pancreatic cancer cells, hence, these doses were utilized for subsequent drug analysis. Similarly, afatinib and erlotinib inhibited the growth of CD18/HPAF pancreatic cancer cells with IC_50_ value of 1 μM and 20 μM concentration, respectively. (Supplementary Fig. S1A and B). We also tested the sensitivity of human telomerase reverse transcriptase immortalized normal human pancreatic ductal epithelial cell line (hTERT-HPNE) to pan-EGFR family inhibitor (Canertinib). As shown in Supplementary Fig. S1C, HPNE cells are more sensitive (IC_50_ of 1.5 μM) to canertinib as compared to pancreatic cancer cells (Supplementary Fig. S1A and B). Canertinib and afatinib treated pancreatic cancer cells showed a decrease in phosphorylation of EGFR at tyrosine 1068, HER2 at tyrosine 1248 and HER3 at tyrosine 1289 residues in pancreatic cancer cells at 5 and 1μM concentration respectively. Interestingly, the expression level of total EGFR and HER2 proteins also decreased with exposure to canertinib and afatinib (Fig. 1A and B). On the other hand, no changes in expression of HER3 and HER4 were detected in the same panel of cell lines for both the drugs (Fig. 1A). Additionally, the effect of canertinib could also influence the localization pattern of EGFR in pancreatic cancer cells. As demonstrated in Supplementary Fig. S2, we observed a decreased membranous localization of EGFR in CD18/HPAF cells treated with canertinib as compared to untreated control cells.

### Pan-EGFR family inhibitors canertinib and afatinib down regulates the expression of MUC4 in pancreatic cancer cells

Previous studies from our lab have shown that MUC4 expression is higher in pancreatic cancer specimens as compared to non-malignant controls [6, 14]. Also, earlier evidence indicates that MUC4 stabilizes HER2 thereby mediating cellular signaling for proliferation and metastasis and imparts resistance to gemcitabine in pancreatic cancer cells [14, 17]. Hence, we next sought to determine the influence of canertinib and afatinib on the expression level of MUC4 along with other EGFR family members. Intriguingly, we found that upon canertinib and afatinib treatment there was a significant reduction in the levels of MUC4 protein (Fig. 1A)

### MUC4 and HER2 co-expression is disrupted by canertinib

As our previous studies have showed MUC4 stabilization and interaction with HER2 [14, 15], we examined whether canertinib could influence the localization pattern of MUC4 and HER2 in pancreatic cancer cells by immunofluorescence analysis. Our results showed decreased membranous co-expression of MUC4 and HER2 in canertinib treated cells (CD18/HPAF and Capan-1) as compared to control cells (Fig. 1B). This result strongly suggests that canertinib can also target MUC4 mucin along with EGFR family members in pancreatic cancer cells.

### Blockade of EGFR mediated STAT signaling inhibits MUC4 mucin protein expression

**Figure 2:**
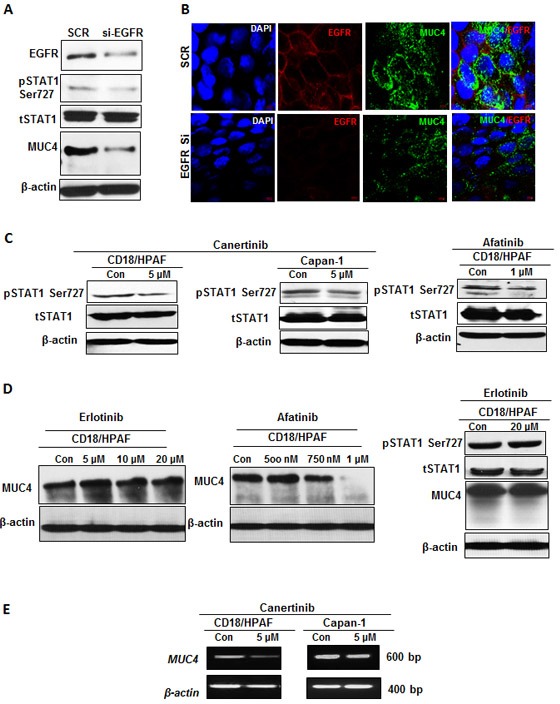
Abrogation of EGFR-STAT1 signaling can contribute to the mechanism of MUC4 regulation (A). Total cellular lysates harvested form CD18/HPAF cells transiently transfected with EGFR specific siRNA or non-targeted (scramble) control was subjected to western blot analysis, with anti-EGFR, anti phospho-STAT1, anti-STAT1, anti-MUC4 and anti-β-actin antibodies. Total EGFR antibody confirms EGFR silencing in the total protein extracts harvested from CD18/HPAF cells, 96 hour following transfection. (B). Confocal analysis of MUC4 and EGFR co-occurrence in EGFR transiently knockdown CD18/HPAF cells. After performing EGFR specific transient knockdown in CD18/HPAF cells for 96h, knockdown cells were further analyzed with co-localization studies for direct MUC4 inhibition through EGFR-STAT pathway. (C). Western blot analysis of total protein lysates harvested from CD18/HPAF and Capan-1 pancreatic cells, untreated or treated with 5 μM concentration of canertinib and 1 μM concentration of afatinib, alone for 24 hour, using phospho-STAT1, total STAT1 and anti-β-actin antibodies. The mechanism by which phospho-STAT1 is inhibited under pan-EGFR inhibitors treatment is directly correlated with MUC4 regulation. (D). Immunoblotting analysis of MUC4 protein expression upon increasing concentrations of erlotinib and afatinib in CD18/HPAF cells. MUC4 protein and its regulatory transcription factor phospho STAT1 is not inhibited even at higher concentration of erlotinib. Whereas, the level of MUC4 protein expression was decreased in a dose dependent manner, with significant inhibition starting at 750 nM and 1 μM concentration of afatinib compared to vehicle treated controls. (E). RT-PCR was performed on pancreatic cancer cells as described in materials and methods. The representative gel images depict decreased *MUC4* transcripts in response to canertinib treatment as compared to vehicle control.

First, we aimed to identify the central mechanism through which pan-EGFR inhibitors inhibits MUC4 protein expression in pancreatic cancer cells. It has been previously shown that EGFR signaling can directly activate STAT and mediate cell migration in esophageal cancer keratinocyte cells [31]. STAT phosphorylation at serine 727 is essential for the transcriptional activity of different genes in cancer cells [32]. Interestingly, our previous study has shown that the MUC4 promoter has STAT1 binding sites and serine phosphorylation of STAT1 (ser727) regulates MUC4 expression in pancreatic cancer cells [33, 34]. Hence, we hypothesized that MUC4 down-regulation following pan-EGFR inhibitor treatment may be mediated through the STAT1 pathway. To determine whether selective silencing of endogenous EGFR led to inhibition of MUC4 mucin expression, we used an EGFR specific siRNA approach in pancreatic cancer cells. As expected, the EGFR siRNA treated cells resulted in decreased phospho-STAT1 (ser727) expression, with no change in total STAT1 expression, which consequently correlated with decreased MUC4 expression (Fig. 2A). Further, densitometry quantification of bands also revealed a 50% reduction in the total EGFR and phospho-STAT1 protein level in the EGFR siRNA transfected cells compared to scramble cells.

In addition, immunofluorescence analysis also showed reduced expression of MUC4 in EGFR specific siRNA transient knockdown cells as compared to scramble siRNA transfected cells (Fig. 2B). In parallel, western blotting was performed on cell lysates obtained from canertinib and afatinib treatment in which STAT1 phosphorylation at serine 727 and tyrosine 701 is significantly down regulated when compared to vehicle control cell lysates (Fig. 2C and Supplementary Fig. S3). Hence, these results imply that pan-EGFR inhibitor inhibits phospho-STAT1 mediated response to regulate MUC4 expression in pancreatic cancer cells, thereby abrogating tumor advancement towards metastasis.

### Inhibition of MUC4 mucin protein via STAT pathway was specific to irreversible TKI but not through reversible TKI

Based on our observations of decreased MUC4 mucin expression upon treatment with irreversible TKIs (canertinib and afatinib) we next sought to investigate whether decreased MUC4 expression is specific to irreversible TKIs or is it a common mechanism for both classes of TKI treatment. To determine the specific effect of small molecule irreversible and reversible TKIs on MUC4 protein expression in pancreatic cancer cells, erlotinib and afatinib were treated with various concentrations-erlotinib (0, 5, 10 and 20 μM) and afatinib (0, 500 nM, 750 nM and 1 μM) for 24 h. We did not observe any reduction or change in the level of MUC4 protein expression in all the doses of erlotinib treatment, as evidenced by western blot analysis. Interestingly, MUC4 protein level markedly decreased as the afatinib concentration increased. The most significant decline in MUC4 protein levels were observed at 750 nM and 1 μM concentration of afatinib as compared to untreated controls. We also assessed the effect of erlotinib on the inhibition of phosphorylation of STAT1 at ser727. Western blot analysis confirmed that the protein level of phospho STAT1, total STAT1 and MUC4 protein is not altered upon erlotinib treatment in CD18/HPAF pancreatic cancer cells for 24h (Fig. 2D). Taken together, our results suggest that MUC4 could be targeted only by irreversible TKIs (canertinib/afatinib) but not by reversible TKI erlotinib. This study thus provides evidence for the therapeutic failure of erlotinib in preventing metastatic events in advanced pancreatic cancer patients.

### Decreased MUC4 protein is partially attributed to decreased MUC4 gene expression by canertinib in pancreatic cancer cells

To further confirm that MUC4 inhibition was a gene transcriptional effect of canertinib treatment, we adopted RT-PCR approach by isolating RNA from pancreatic cancer cells treated with canertinib and its corresponding vehicle control. Consistent with our MUC4 protein expression pattern, the level of MUC4 mRNA was observed to decrease in canertinib treated pancreatic cancer cells (CD18/HPAF and Capan-1) (Fig. 2E). This result suggests that the decreased MUC4 protein expression following canertinib exposure is a consequence of both the effect on MUC4 regulation and impaired *MUC4* gene expression in pancreatic cancer cells.

### Inhibition of ERK1/2 phosphorylation and cell cycle key regulator expression by canertinib or afatinib decreases pancreatic cancer cell survival

**Figure 3:**
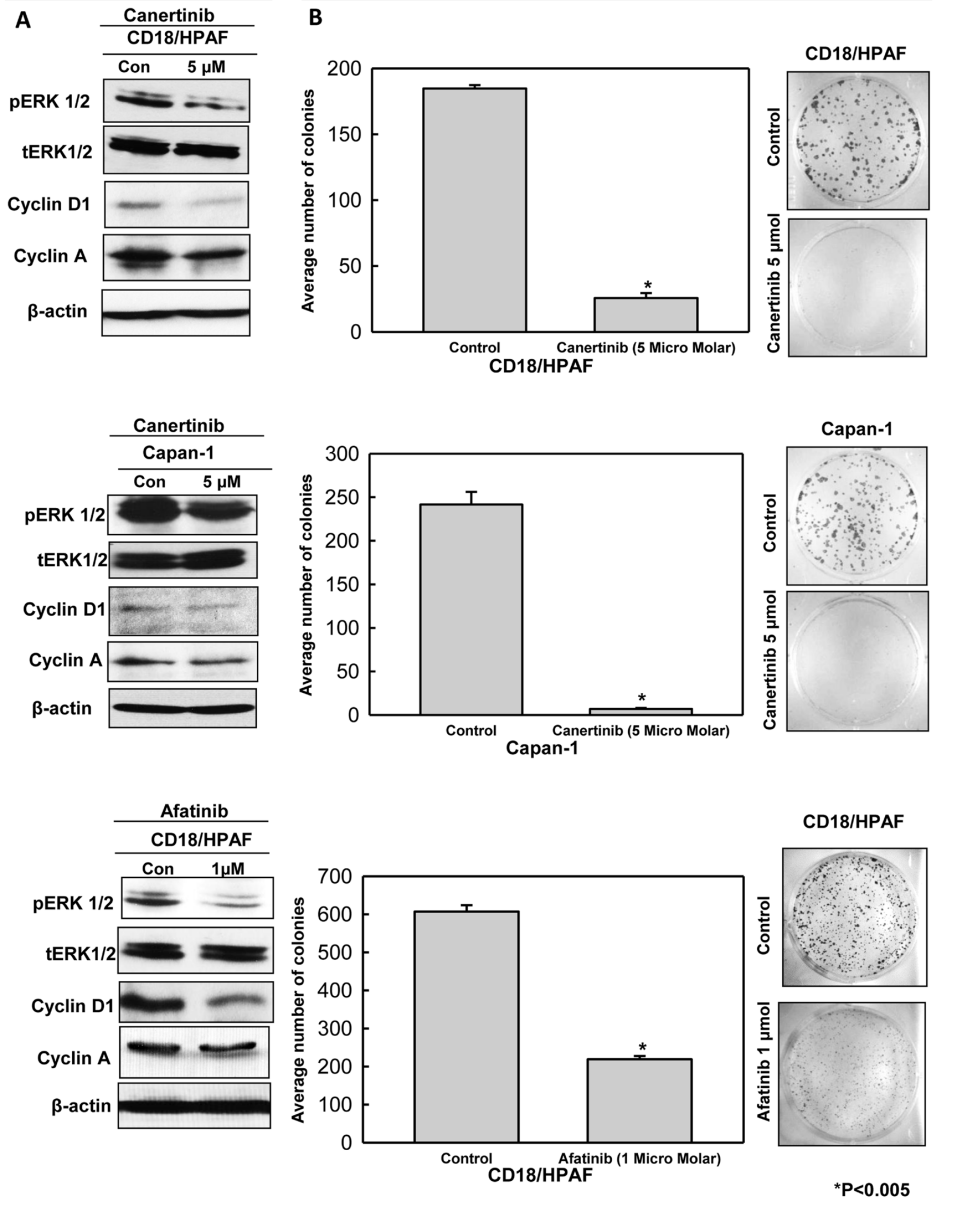
Canertinib and afatinib induced down regulation of cell survival and cell cycle related protein is partly responsible for decreased cell proliferation and survival of pancreatic cancer cells (A). Treatment of pancreatic cells (CD18/HPAF and Capan-1) resulted in down regulation of major proteins responsible for cellular proliferation and cell cycle regulation. Immunoblotting analysis with specific antibodies toward phospho-ERK1/2 and Cyclin D1 and Cyclin A was carried out on whole cell lysates of pancreatic cancer cells incubated in the presence or absence of canertinib and afatinib for 24 hours. The total ERK remains unchanged in control and treatment conditions. Beta actin was used as the loading control. (B). Influence of canertinib and afatinib on cell survival and viability was functionally determined by colony forming assay. Colony forming assay was done in CD18/HPAF (0.1-0.3×10^4^) and Capan-1(0.1×10^4^) pancreatic cancer cells with indicated concentrations of canertinib, afatinib and DMSO treatment. Six well plates were seeded with above mentioned pancreatic cancer cell density in complete medium. After 48 hours of incubation, the cells were washed with PBS and supplement with inhibitors or vehicle in complete medium (10% DMEM). After a period of 10 days of incubation the cells were fixed with 100% ice cold methanol and stained with 0.4% crystal violet in methanol. Representative images show significant reduction in the number of colonies in the canertinib and afatinib treated cells as compared to vehicle or untreated control cells.

To provide a comprehensive analysis of the effects of pan-EGFR inhibitor treatment on cell survival, we performed a Western blot analysis of control and inhibitors treated lysates for molecules that participate in various signaling pathways. As shown in Fig. 3A decreased phosphorylation of ERK1/2 in pancreatic cancer cell lines was observed with no change in the corresponding total ERK1/2 level with both pan-EGFR inhibitors treatment. Furthermore, we examined the effect of canertinib and afatinib on cell cycle related proteins Cyclin D1 and Cyclin A by Western blot analysis. As shown in Fig. 3A, the level of Cyclin D1 and Cyclin A decreased after 24 hours of treatment with both drugs. Previous studies have shown that altered expression of MUC4 can drive cell cycle protein alterations [14]. We observed that canertinib and afatinib treatment of pancreatic cancer cells resulted in decreased cell cycle protein expression. Thus, canertinib and afatinib could involve either independently or MUC4 dependent regulation of pancreatic cancer cell growth and cell cycle alteration. Further, to evaluate the effect of canertinib and afatinib on cell survival, colony formation assay was performed in CD18/HPAF (0.1-0.3×10^4^ cells/well) and Capan-1 (0.1×10^4^) pancreatic cancer cells. In the cell lines tested, significant reduction in the number of colonies was observed in the wells treated with canertinib and afatinib, suggesting that these drugs might have a dual effect on cell proliferation and cell survival (Fig. 3B).

### Canertinib and afatinib inhibits migration of pancreatic cancer cells

**Figure 4:**
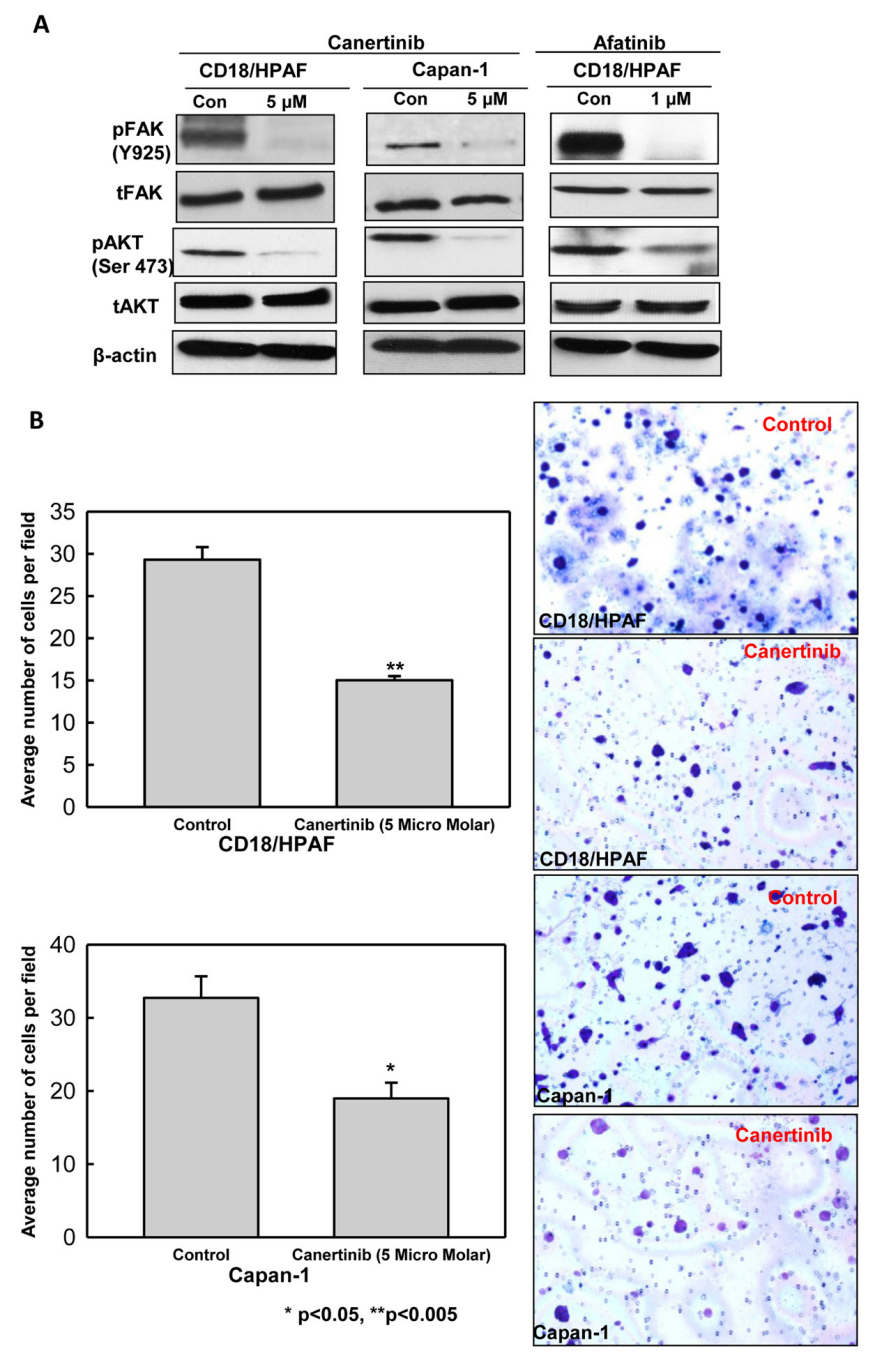
Canertinib and afatinib inhibits migration of pancreatic cancer cells (A). Western blot results shows that canertinib and afatinib treatment affects the phosphorylated form of FAK at tyrosine 925 and phospho-AKT at serine 473 with no change in the total FAK and AKT levels when compared to control cells. (B). Migration of pancreatic cancer cells in presence and absence of Canertinib. Trans-well migration assay was performed with canertinib treatment in CD18/HPAF and capan-1 cells. 10% fetal bovine serum medium was used as a chemo attractant. After 24 hour of incubation, the cells remaining above the insert membrane were removed by gentle scraping with a sterile cotton swab. Cells that invaded through the bottom of the insert were fixed and stained with crystal violet. Invading cells on representative sections of each membrane were counted under light microscopy. Bar data summarizes the average number of migrated cells per field between the control and treatment pancreatic cancer cells. Representative images of pancreatic cancer cells migration towards a concentration gradient of chemo attractant set up in the transwell filter.

**Figure 5:**
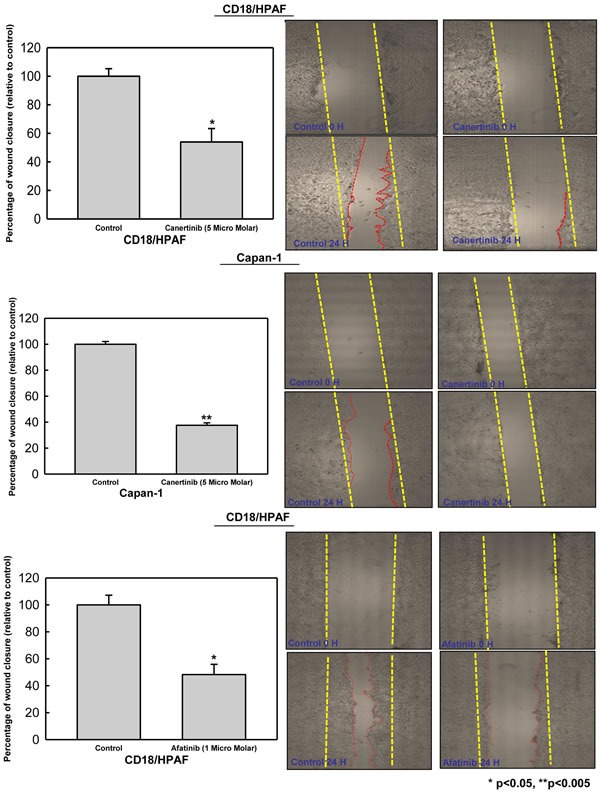
Canertinib and afatinib inhibits motility of pancreatic cancer cells CD18/HPAF and Capan-1 cells were trypsinized, counted and seeded at a density of 2×10^6^ cells in 60-mm Petri dishes and kept in 10% DMEM overnight. To determine the effect of canertinib and afatinib upon wound closure artificial wounds were created in 90% confluent cells and after 24 hour the cells were treated with vehicle DMSO (0.01%), canertinib 5 μM and afatinib 1 μM in complete medium. Images were taken at 0 and 24 hour in both control and inhibitor treated cells and cells migrated in the wound were measured by measuring the distance of migration The bars represents the percentage of wound closure between the control and treatment with significant p value less than 0.05 in CD18/HPAF (for both inhibitors) and p value less than 0.005 in capan-1 cells. Representative images of wound areas obtained at 0 and 24 hours before and after addition of canertinib and afatinib in pancreatic cancer cells. The red dotted lines depict the area of wound closure between the treatment and control cells.

FAK pathway is essential for the motility or migration of cancer cells, which was correlated with MUC4 expression in pancreatic, ovarian and breast cancer cells [11, 15, 35]. We have observed the decreased level of activated FAK (Y925) in canertinib and afatinib treated pancreatic cancer cells compared to control cells (Fig. 4A). We also examined the sensitivity of pan-EGFR inhibitor on MUC4 negative background. As shown in Supplementary Fig S4, MiaPaCa-1 cells (A MUC4 negative pancreatic cancer cells) were treated with indicated concentration of canertinib and analyzed for pHER2, total HER2, MUC4 and pFAK protein expression. Canertinib treatment resulted in marginal decrease of pFAK in MiaPaCa-1 cells, whereas complete inhibition of pFAK was achieved in MUC4 positive pancreatic cancer cells (CD18/HPAF and Capan-1) (Fig. 4A). Trans-well motility and wound healing assays were used to measure the migration capacity of human pancreatic cancer cells under various *in vitro* conditions. Canertinib treatment resulted in a significant reduction in the migratory potential of CD18/HPAF and Capan-1 cells, as evidenced by Trans-well migration assay (Fig. 4B). Additionally, treatment with Canertinib and afatinib at the indicated concentration also resulted in impaired migration of pancreatic cancer cells to migrate in to the wounded area (Fig. 5). Previous reports have shown that FAK and AKT are dual kinases that play a key role in cancer cell progression specifically in metastasis. It is well established that cancer cell adhesion is dependent on AKT dependent activation of FAK [36]. Our results showed that both canertinib and afatinib significantly inhibited AKT-phosphorylation at Ser-473 in pancreatic cancer cell lines whereas no change in the levels of total AKT was observed (Fig. 4A). Thus, canertinib and afatinib inhibits the migration of pancreatic cancer cells by inhibition of AKT pathway and through a FAK dependent mechanism.

### Effective inhibition of tumor growth and metastasis with canertinib: *In vivo* pancreatic cancer orthotopic model

**Figure 6:**
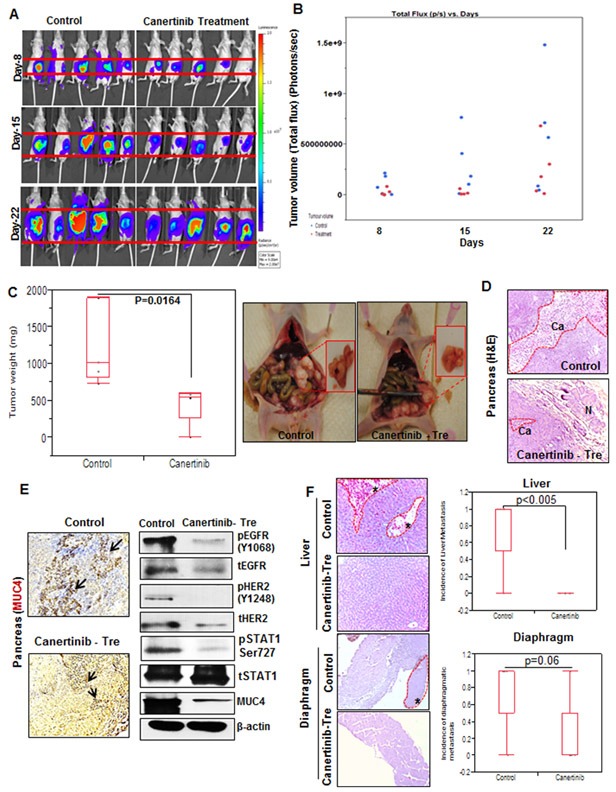
*in vivo* evaluation of canertinib using an orthotopic mouse model of pancreatic cancer (A). Six weeks old female athymic nude mice were orthotopically implanted with luciferase-tagged CD18/HPAF cells in pancreas. The mice were randomized into two groups: group1 received an i.p injection of PBS as a vehicle control and group 2 was given canertinib orally 5 days a week with 5 mg/kg/day dissolved in PBS. Bioluminescent images of control and animals under treatment were obtained at regular intervals at 0, 8, 15 and 22 days using Xenogen optical *in vivo* imaging system (IVIS) located at the UNMC animal facility. Representative animal images show significant reduction of tumor volume at day 8, 15 and 22, as compared to the control group. (B). Scatter plot was drawn based upon emitted photons captured using IVIS machine in the control and treatment groups. Total efflux was calculated by measuring the photons emitted per second from each animal. All the data's were obtained by normalizing the values of photons emitted in animals of 22^nd^ day (both control and canertinib treatment with 15^th^ and 8^th^ day of treatment schedule. The plot shows a significant decrease in the tumor volume in canertinib treated mice compared to PBS treated control mice. (C). All mice were sacrificed after 4 weeks of treatment and the tumor volume weighed. The final weight of each of the tumors obtained from both the groups are illustrated by a box plot, each mouse tumor is represented as a dot in the box plot, p=0.0164. The representative images of mice pancreas after euthanasia. Both the group mice were found to develop tumor growth in the pancreas after orthotopic transplantation. However, mice receiving canertinib treatment showed significant decreased tumor volume (right) compared to controls (left). (D). Excised and processed orthotopic pancreatic tissues section were stained with hematoxylin and eosin and images were taken at 20X magnification. (E). Primary tumors (pancreas) from the control and inhibitor treated tissues sections were checked by immunohistochemical analysis for MUC4 expression using anti-MUC4 (8G7, 1:2500) and counterstained with hematoxylin. The analysis revealed enhanced MUC4 staining in the control group than canertinib treated mouse pancreas. Whole cell lysates were prepared from orthotopically implanted pancreas isolated (from control and treatment mice) during sacrifice. Cell lysates were separated by 10% SDS-PAGE and analyzed using phospho-EGFR, phospho-HER2, phospho-STAT1, MUC4 specific antibodies. Immunoblots analyzed for phosphorylation of EGFR, HER2 and STAT1 were striped and re-probed with their respective total proteins. Beta actin was used as the internal loading control. (F). Impact of pan EGFR family small molecule inhibitor canertinib on controlling mice pancreatic tumor metastasis. Representative images of metastatic tissue sections (liver and diaphragm) showing histopathological changes in the control and canertinib treatment mice. Box plot shows a decrease in the incidence of metastasis to the liver (p<0.005) and diaphragm (p=0.06) upon treatment with canertinib as compared to vehicle treated control mice. The metastatic nodules were indicated with asterisks in the liver and diaphragm sections.

On the basis of our *in vitro* experiments we sought to test the effects of canertinib on tumorigenicity and metastasis using the *in vivo* pancreatic cancer orthotopic model (CD18/HPAF luciferase tagged pancreatic cancer cells). The dosage and treatment schedule for the orthotopic mouse model system were illustrated in Supplementary Fig. S5A and we observed a significant growth inhibitory effect (non-invasively via imaging) in the pancreatic tumor-bearing mice treated with canertinib compared to control mice (Fig. 6A). The images obtained from the CD18/HPAF luciferase tagged cells were further quantified as per the treatment schedule mentioned in the material and methods section and the emitted photons were normalized with in a range of 9.00e^4^ to 2.00 e^7^. The scatter plot images revealed the maximum efficacy of canertinib drug treatment in inhibiting cancer growth *in vivo*, at each time point (Fig. 6B). An average tumor weight of 1887 ± 480 mm^3^ in the control group was observed as compared to 751 ± 526 mm^3^ in the drug treated group (*P*=0.0164) (Supplementary Table 2 and Fig. 6C). In order to evaluate the pathological effects of canertinib on *in vivo* model system the excised mouse primary pancreatic tissues sections were subjected to hematoxylin and eosin staining. As shown in Fig. 6D, canertinib oral treatment of 5 days on and 2 days off schedules with 5 mg/kg/day in PBS for 3 consecutive weeks leads to significant reduction of tumor growth of orthotopically implanted mouse pancreas. Further, immunohistochemical and western blot analysis was performed on the tumor tissues isolated from the control and treatment groups. The immunohistochemical analysis showed a reduced expression of MUC4 in inhibitor treated orthotopic tissues as compared to control sections. Similarly, immunoblot analysis also revealed a reduced MUC4, pEGFR, pHER2 and phospho-STAT1 along with their respective total proteins under treatment conditions compared to controls. These observations are consistent with our *in vitro* assays as a markedly reduced expression of EGFR, HER2 and MUC4 proteins were observed (Fig. 6E).

Interestingly, we found a significant difference in the incidence of metastasis between the control and canertinib treated groups in various organs such as liver (p <0.02), spleen (p <0.02), ovary (p <0.04), diaphragm (p=0.06) and intestine (p<0.04) (Fig. 6F and Supplementary Fig. S5B). The histopathological changes were evaluated using hematoxylin and eosin staining in the primary pancreatic tumor tissues and the metastatic organ tissues isolated from the control and inhibitor treated mice. Tumor metastasis to distant organs such as the liver, stomach, ovary, diaphragm and colon was clearly inhibited by canertinib treatment (Table 1 and Supplementary Fig. S5B).

**Table 1:** Effect of canertinib in controlling incidence of metastasis by CD18/HPAF pancreatic cancer cells to various organs in orthotopic nude mice

Groups	Metastatic sites										
	Liver	Spleen	Intestine	Stomach	Ovary	Diaphragm	Peritoneum	Colon	Ascetic fluid	Mesenteric lymph node	Lungs
Control	4/5	5/5	3/5	2/5	4/5	4/5	3/5	3/5	2/5	1/5	0/5
Canertinib	0/5	1/5	2/5	0/5	0/5	0/5	2/5	0/5	0/5	0/5	0/5
P value	0.016	0.016	0.07	0.177	0.016	0.066	0.24	0.07	0.177	0.37	-

## DISCUSSION

Pancreatic cancer is the most lethal form of cancer with a very high incidence of distant metastasis due to aberrant expression of tumor antigens [37, 38]. We explored for the first time the novel role of pan-EGFR family inhibitors in reducing the metastatic potential of pancreatic cancer, with major emphasis on MUC4 inhibition using multiple cell lines and an orthotopic mouse model. Most pancreatic carcinomas (95%) aberrantly express EGFR, HER2 and HER3 receptors and their cognate ligands, promoting constitutive activation of EGFR family members that result in pancreatic cancer cell proliferation [39, 40]. We and others have shown that MUC4 mucin is aberrantly overexpressed in various cancers including pancreatic cancer [5, 9, 11, 12, 14, 41]. Furthermore, MUC4 mucin overexpression correlates with its capacity to potentiate tumor invasion and metastasis in pancreatic cancer [11]. We have further validated the oncogenic role of Muc4 in the spontaneously developing pancreatic cancer mouse progression model [42]. Thus direct or indirect inhibition of MUC4 mucin would be an effective strategy to suppress MUC4 mediated cancer cell invasion and metastasis to distant organs.

It has been reported previously that EGFR is likely to activate STAT in a manner unique from other STAT activation mechanisms in other malignancies [43]. Further, STAT1 protein is reported to be expressed in 88% of primary pancreatic cancer patients [44], which implies the clinical significance of STAT1 in pancreatic cancer. Since our previous study has demonstrated that the MUC4 promoter has STAT1 binding sites and regulates MUC4 expression in pancreatic cancer cells [33], we investigated whether the levels of phosphorylated STAT1 could influence MUC4 in pancreatic cancer cells treated with the pan-EGFR inhibitors. To prove this we transiently inhibited endogenous EGFR using siRNA in pancreatic cancer cells and, as expected, transient knockdown of EGFR expression led to inhibition of phospho-STAT1 thereby down regulating MUC4 mucin protein expression. In addition to the transfection studies, treatment of pancreatic cancer cells with canertinib and afatinib at the indicated concentrations also resulted in significant inhibition of phospho-STAT1 and decrease of MUC4 mucin protein expression. Down-regulation of MUC4 expression in canertinib and afatinib treated cells are further corroborated by down regulation of p-STAT1 (Ser727 and Y701), a transcriptional activator of MUC4 [45]. To further confirm the EGFR mediated MUC4 mucin down-regulation, confocal microscopy analysis of EGFR and MUC4 was also performed in EGFR knockdown CD18/HPAF cells, which showed similar observations as that of Western blot studies. Thus, confirming that inhibiting the EGFR mediated STAT pathway can block MUC4 mucin regulation. On the other hand, we investigated the significance of EGFR specific reversible inhibitor erlotinib on MUC4 downregulation. Interestingly, erlotinib was unable to attenuate MUC4 protein level in pancreatic cancer cells which could be possibly due to the unaltered phosphorylation status of STAT1 (Ser 727). Thus, treatment with erlotinib appears to be ineffective in controlling MUC4 mucin protein in pancreatic cancer.

Indeed, *in vitro* addition of canertinib and afatinib to pancreatic cancer cells also affected cell proliferation and survival. Our results show that there is a decrease in the number of colonies and consequently as significant reduction of phospho-ERK1/2, CyclinD1 and CyclinA protein levels in inhibitors treated cells. Further canertinib and afatinib significantly contributes towards the inhibition of migration or metastasis of pancreatic cancer cells. These were confirmed by transwell migration and wound healing assays. The ability of these inhibitors to modulate FAK phosphorylation adds evidence on the effect of canertinib towards FAK mediated cell migration. Finally, the *in vivo* antitumor efficacy of pan-EGFR inhibitor canertinib was evaluated using pancreatic tumor bearing mice. Our results show that canertinib pharmacologically reduces tumor burden by inhibiting EGFR family members and prevents metastatic events by down-regulating MUC4 mucin protein.

Briefly, canertinib and afatinib are irreversible pan-EGFR TKIs that have been shown to be effective in various cancers, but its therapeutic efficacy was not explored in pancreatic cancer in the context of MUC4 mucin. Further, the reasoning behind our concept of utilizing the effect of pan-EGFR inhibitor in abrogating MUC4 mucin in pancreatic cancer are as follows: (i) MUC4 is oncogenic and is overexpressed in pancreatic cancer [14, 37] and (ii) MUC4 potentiates invasion, migration and metastasis of pancreatic cancer cells by interacting with EGFR family protein HER2 [7, 12, 14, 15]. Thus, the indirect pharmacological inhibition of MUC4 via canertinib or afatinib could result in developing pan-EGFR inhibitors as a molecular therapeutic agent against advanced pancreatic cancer. Notably, we were also able to show that these changes are also associated with a modest decrease in expression of endogenous MUC4 total RNA, which is sufficient to impair its downstream effectors such as FAK, AKT and ERK and result in impaired motility, proliferation and metastasis of pancreatic cancer cells.

Furthermore, in our study, we have demonstrated that canertinib and afatinib could influence pancreatic cancer cell migration, as FAK is regarded as the initiator of cell migration and cancer metastasis [46]. Additionally, cancer cell adhesion is shown to be mediated by AKT and FAK interaction and FAK was shown to be upstream of AKT activation. In addition, a previous work of our group have demonstrated that ectopic overexpression of *MUC4* in ovarian cancer cells leads to up-regulation of FAK mediated downstream signaling [47]. Notably, in our study we have demonstrated the role of pan-EGFR inhibitors in limiting the migration of pancreatic cancer cells through FAK mediated pathway. On the other hand, mitogen-activated protein kinases (MAPKs), including extracellular signal-regulated kinase 1/2 (ERK1/2), c-Jun NH2-terminal kinase (JNK) and p38, are a family of signaling molecules that participate in cell survival [48]. Previous reports have shown that canertinib treatment causes cytotoxicity by the induction of MAPK phosphorylation through ROS generation in several cancer cell lines [25]. In this study, we found that canertinib and afatinib decreases ERK1/2 phosphorylation in pancreatic cancer cells, possibly through ROS generation. Another important finding in our current study is that these pan-EGFR inhibitors significantly inhibit phosphorylation of AKT at Ser-473 in pancreatic cancer cells. Similar observations have been previously reported in human esophageal cancer cells [23]. In addition, we found that treatment with pan-EGFR inhibitors also resulted in down regulation of cell cycle key regulators such as Cyclin D1 and Cyclin A, implicating that down regulation of ERK1/2 and AKT pathways affects cell cycle progression. Moreover, a recent study has exemplified the novel function of AKT which promotes FAK auto-phosphorylation for cell migration and motility [49]. Together these results indicate that canertinib affects both the proliferative (ERK) and survival (AKT) pathways.

It has also been described earlier that the irreversible pan EGFR family inhibitor canertinib can exert its anti-tumor effect both *in vitro* and *in vivo* in various malignancies such as breast, ovarian, colon, skin and esophageal cancer [22-24, 26, 50]. It is important to note that through our *in vivo* study, we were able to get tumor growth inhibition at a much lower concentration of 5 mg/kg/day in PBS. In addition we also did not observe toxicity in the orthotopic *in vivo* model system. Treatment with canertinib at 40 mg/kg/day resulted in 69% of tumor reduction as compared to controls while 80 mg/kg/day resulted in growth inhibition with wide-spread antitumor activity in mouse xenograft models [25, 26]. This short term treatment of canertinib (22 days) decreased tumor growth and micro and macro metastasis in our pancreatic cancer xenograft model. Canertinib specifically blocks phosphorylation and activation of EGFR and HER2 and expression of MUC4 and its associated potential of metastasis initiation *in vivo*. Thus, these preclinical data suggests that partial abolishment of the MUC4 mucin mediated signaling axis along with inhibition of EGFR family members could be a more effective approach to combat pancreatic cancer as it inhibits the crosstalk between multiple pathways and could result in a highly efficient therapy when used in combination with primary care of pancreatic cancer. Previously, the US FDA has approved the combination of erlotinib with gemcitabine for the treatment of locally advanced PC patients [2]. However, there are certain front line benefits of canertinib and afatinib (irreversible TKI), which makes it a far superior agent than EGFR specific reversible TKI erlotinib in pancreatic cancer therapy. Briefly, the advantages are as follows: (i) irreversible second generation TKI targets all the EGFR family proteins whereas, reversible TKI targets EGFR alone; (ii) Irreversible TKI prevents heterodimerization of HER3 with other EGFR family proteins, bur reversible TKI cannot ; (iii) Sustained clinical impact hence, less dosage is required for administration (5 and 1 μM of canertinib and afatinib) whereas the dosage to be administered is high, as evidenced by our study (erlotinib 20 μM); and (iv) Prevents compensatory activation of other EGFR family members, in contrast reversible TKI activates alternative HER3 mediated signaling making it difficult to combat the disease. Canertinib and afatinib differs in their half-life and effective dosages. Canertinib have half-life of 2-4h whereas, afatinib has 30-40h [51, 52]. Similarly based on our *in vitro* studies, the concentration of canertinib and afatinib required for the abrogation pancreatic cancer cell growth is demonstrated as 5 μM and 1 μM, respectively.

**Figure 7:**
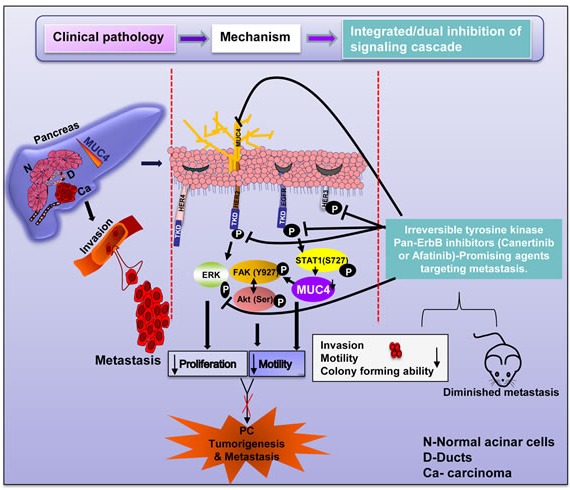
Overall representation of our study hypothesis and the mechanism by which pan EGFR family irreversible inhibitors (canertinib and afatinib) induces down regulation of MUC4 and EGFR family proteins to attenuate proliferation, migration and metastasis of pancreatic cancer The entire schematic diagram is divided into three sections: (i) Clinical pathology which illustrated that mucins are aberrantly overexpressed in various cancers including pancreatic cancer and specifically MUC4. The absence of MUC4 expression in normal pancreas differentiates MUC4 from other mucins to be considered as one of the marker which is abnormally expressed in pancreatic tumor tissue as well as tumor cell lines. Furthermore, the enhanced MUC4 regulation in primary tumor initiates complex signaling events, which culminates the process of invasion and distant organ metastasis. (ii) Mechanism- Our *in vitro* culture methods have evaluated the mechanism and dual role of pan-EGFR family TKIs in controlling the EGFR family members as well as mucin specifically MUC4 mediated inhibition of cellular effects.(iii) Integrated/dual inhibition-Extending from the *in vitro* methods to *in vivo* system. Following orthotopic implantation of pancreatic tumor cell line in mouse pancreas, 100% (5/5) of mice receiving canertinib orally showed significant decrease in tumor weight. Notably, canertinib was more effective in controlling distant organ metastasis. These results are in conclusion that pan-EGFR family inhibitors are effective in controlling MUC4 mediated pancreatic cancer pathogenesis advancing towards metastasis, apart from its regular EGFR family members mediated proliferation control.

Based on these findings, we conclude that pan-EGFR inhibitor (canertinib or afatinib) will specifically down regulate EGFR and HER2 and result in an anti-proliferative effect in pancreatic cancer cells. It has also been shown that canertinib can down regulate MUC4, a molecule that contributes towards pancreatic cancer aggressiveness. Additionally, we explored the central mechanism of EGFR; STAT1 mediated MUC4 mucin regulation through siRNA approach. Previous studies have shown that HER3 and HER4 are regulated by heterodimerization of EGFR or HER2. Hence, the change in the total level of EGFR and HER2 in response to canertinib and afatinib treatment, predicts a possible inactivation of HER3 and disabling of HER4 mediated signaling events such as proliferation and apoptosis in pancreatic cancer cells. Thus, these results conclude that canertinib and afatinib may also target heterodimerization mediated cellular response which could be a possible mechanism for the failure of EGFR based targeted therapy. More importantly, our *in vivo* studies using canertinib was also promising in abrogating liver, diaphragm and spleen metastasis, which are the most common sites of metastasis in pancreatic cancer patients. Overall, our results for the first time demonstrate that pan-EGFR inhibitors (canertinib and afatinib) treatment can modulate EGFR family members and their downstream signaling along with MUC4 mucin inhibition, thereby significantly contributing towards inhibiting pancreatic cancer cell survival and metastasis (Fig. 7). This study does shed light on integrated treatments, i.e. chemotherapy combined with targeted therapies, and could pave the way for personalized therapy which could lead to a novel treatment and therapeutic strategy for combating lethal pancreatic cancer. This study highlights the urgent need to develop molecular targeted therapy in combination with chemotherapeutic agents to inhibit mucins and its associated signaling molecules in pancreatic cancer. In summary, our results demonstrate that canertinib and afatinib as attractive drugs to inhibit the pancreatic cancer growth and metastasis when it is used along with other traditional cytotoxic agents like gemcitabine or abraxane in clinical studies.

## MATERIALS AND METHODS

### Cell lines and reagents

CD18/HPAF, Capan-1 and MiaPaCa-1 pancreatic cancer cells and human immortalized pancreatic ductal epithelial cells hTERT-HPNE were purchased from ATCC and were maintained as per the ATCC recommendations and based on our previous publications [11, 12, 14, 45]. The pan EGFR family members specific and EGFR specific inhibitors used in this study (Selleck Chemicals, TX, USA) were dissolved in either DMSO (canertinib and erlotinib) or in PBS (afatinib) and stored at −20°C as per the manufacturer's recommendation.

### Growth inhibition assay

For the growth inhibition assay, 3×10^4^ pancreatic cancer cells and human telomerase reverse transcriptase immortalized normal human pancreatic ductal epithelial cells hTERT-HPNE were plated into flat-bottomed 96-well plates (Costar, Corning, NY). After 24 hours, various concentrations of canertinib (0, 0.5, 1, 2, and 5 μM), afatinib (0, 500nM, 750 nM 1 and 2 μM) and erlotinib (0, 5, 20 and 40 μM) were added to the pancreatic cancer cells and they were incubated for an additional 24 hours. Subsequently, MTT assay was performed as per the standard procedure [45].

### Transfection studies

CD18/HPAF pancreatic cancer cells (0.5 ×10^6^) were seeded into 60 mm culture dishes. After 24 hour, cells were transiently transfected with a pool of four siRNA oligonucleotides specific for human EGFR (100 pmol) (MU-003114-01-002, siGENOME SMART pool, Dharmacon Research, Inc., Lafayette, CO) using DharmaFECT 1 transfection reagent as per manufacturer's instructions for 96 hours. Non-targeting (siRNAs) oligonucleotides were used as transfection control. The transfected cells were harvested 96 hours after transfection. The lysates were collected and analyzed for EGFR, phospho-STAT1 and MUC4 protein expression by western blotting analysis.

### Confocal immunofluorescence microscopy

The methods employed for confocal analysis were previously described from our group [14, 15]. Pancreatic cancer cells (5×10^4^) were grown on sterilized cover slips for 24 hours and treated with appropriate DMSO (vehicle control), canertinib and afatinib inhibitors at 5 and 1 μM concentrations and further incubated for 24 hours. Briefly, cells were incubated with the following antibodies to detect their localization pattern: anti-MUC4, anti-HER2 and anti-EGFR (Supplementary Table 1). Following primary antibody incubation, cells were incubated with FITC conjugated anti-mouse (MUC4 & EGFR), Texas red-conjugated anti-rabbit (HER2) and DAPI for nuclear staining. Advanced laser scanning confocal microscopy was performed using an LSCM 710 camera (Carl Zeiss GmbH, Jena, Germany) [14, 15, 45].

Similarly, confocal analysis was also performed in EGFR-siRNA and scramble-siRNA transfected cells after 96 hours. Transient transfection and confocal immunofluorescence analysis were performed as described previously [15, 45]. Anti-EGFR and anti-MUC4 antibodies used in this study were briefly described Supplementary Table 1. Appropriate secondary antibodies such as Goat anti-Rabbit IgG coupled with Alexa Flour red (568) for EGFR and Alexa Flour green (488) Goat anti-Mouse for MUC4 were used for immunofluorescence detection of pancreatic cancer cells.

### Colony forming assay

Colony forming assays were performed as described previously [53]. Briefly, cell survival was determined by plating approximately 0.1-0.3×10^4^ (CD18/HPAF and Capan-1) cells/well in a six-well plate in triplicates. After 24 hours of incubation with complete media, 5 and 1μM concentration of canertinib and afatinib were added and further incubated for 10 days at 37°C in a humidified atmosphere of 5% CO_2_. The control cells were incubated with 0.01% of DMSO. The cells were harvested and fixed with 100% methanol, stained with 0.4% crystal violet in methanol and colonies were counted using Quantity One software (Bio-Rad, Richmond, CA, USA) [53].

### Migration and wound healing assay

The transwell migration and wound healing assays in pancreatic cancer cells under various treatment conditions were performed with slight modifications as described previously [11, 14, 35, 45]. Trans-well migration assay were performed using modified boyden chamber consisting of a cell culture inserts with 8-μm pore size, polyethylene terephthalate track-etched membrane (Falcon#353093) of six well format seated into a six well cell culture plates. Briefly, pancreatic cancer cells CD18/HPAF and Capan-1 were counted and suspended in a serum free DMEM at a density of 1×10^6^/well. The bottom chamber of the six well plate were added with 2.0 ml of 10% FBS (Chemotaxis movement) in DMEM. The corresponding drug canertinib (5 μM) or 0.01% DMSO (control) were also added to cell suspension in the serum free medium and seeded into the upper chamber of insert. The migrations of cells were allowed for 24 hour in a humidified atmosphere at 37°C with 5% CO_2_. Cells that invaded through 8-μm pores into the Trans-well cell culture chamber were fixed and stained with Diff-Quick cell staining kit (Dade Behring, Inc.). The pancreatic cancer cells that migrated towards the lower surface of the culture chamber were counted in 12 random fields under a light microscope. Similarly, a wound healing assay was performed to study the pancreatic cancer cell migration under canertinib and afatinib treatment conditions. Pancreatic cancer cells CD18/HPAF and Capan-1 were seeded at a density of 2×10^6^/well supplemented with 10% FBS in DMEM. After overnight incubation and obtaining confluent (>90%) cultures, an artificial wound was created using a 200μl sterile pipette tip. The culture plates were washed with PBS to remove the damaged and detached cells. Cells were treated with drugs such as canertinib (5 μM), afatinib (1 μM) and 0.01% DMSO vehicle control were added into the DMED medium containing 10% FBS for 24 hour. Images were taken at 0 and 24 hours at 10X magnification using Accu-scope microscope assisted with Moticam 580 digital camera. The wound closure was calculated by measuring the distance between two edges of wounds using Image J software. Twelve independent areas in the wound per image were counted in arbitrary units using a straight line tool in the image j software. The mean distance between the wound edges at twelve independent areas of the control and treatment at 0 and 24 hour was calculated and the arbitrary units were converted into percentage as follows. % of wound remaining = (Mean area of measurement at 24 h/Mean area of measurement at 0h) multiplied by 100. Further, the percentage of wound closure was calculated as: 100% - % of wound remaining [11, 14, 35, 45].

### RNA isolation and reverse transcription PCR analysis

RNA isolation and PCR amplification conditions were followed as described previously [12]. RNA was isolated using the QIAGEN RNeasy mini kit (Qiagen, Valenica, CA, U.S.A.) and its concentration was determined using a NanoDrop ND 1000 Spectrophotometer. cDNA was synthesized using 2 μg RNA, oligo(dT)_18_ primer, and Super Script II RNase reverse transcriptase (Invitrogen). PCR was performed for MUC4 using the primer sequences: Forward primer 5′-CGCGGTGGTGGAGGCGTTCTT-3′ and reverse primer 5′-GAAGAATCCTGACAGCCTTCA-3′. β-actin was used as an internal control [12].

### Immunoblotting

Western blot analysis was performed as described previously [14, 15]. Briefly, pancreatic cancer cells were treated for 24 hour with: (i) complete medium+0.01% DMSO (control); (ii) 5 μM canertinib; (iii) 1 μM afatinib. Whole-cell lysates having approximately 40 μg of proteins were resolved on 10% SDS–PAGE and subjected to western blotting using list of antibodies described in supplementary Table 1. For the separation of high molecular weight MUC4 protein we will resolve 40 μg of proteins lysates on a 2% SDS agarose gel. After appropriate secondary antibody incubation the bands were visualized using enhanced chemiluminescence (ECL) method (Thermo scientific).

### *In vivo* mice model studies

The study was performed based on IACUC protocol approved by the UNMC Animal Ethics Committee and as per the NIH guidance of the care and use of laboratory animals. Four to six week-old female athymic nude mice (n=5 for each group) were obtained from NIH and maintained in pathogen free conditions in the institutional animal facility. To establish tumor growth in mice, 0.5×10^6^ of CD18/HPAF luciferase tagged cells, suspended in 100 μl phosphate-buffered saline (PBS) were orthotopically implanted into the mouse pancreas. Initially the tumors were allowed to grow for 10 days without treatment and animals were randomized into two groups of 5 animals each based on the fluorescence image quantification identified using Xenogen optical *in vivo* imaging system (IVIS). Group 1 acted as control and was given PBS as vehicle control; group 2 was given canertinib alone. The treatment schedules were as follows: canertinib was given orally 5 days a week with 5 mg/kg/day in PBS and control mice received intraperitoneal (i/p) injection of PBS as vehicle control. Further, these animals were (i/p) injected with luciferin and were subjected to imaging using IRIS machine, at time points 0, 8, 15 and 22 days of treatment. The emitted photons were calculated using Living image software version 4.4. After four weeks of treatment, all the animals were sacrificed by aspiration with CO_2_; tumors were removed and weighed. Metastatic sites were observed and the respective tumor and metastatic organs were collected for immunohistochemistry and protein analysis.

### Histological, immunohistochemical and immunoblot studies

To analyze histological changes associated with canertinib treatment along with control PBS treatment mice, we fixed the tumor specimens in 10% buffered formalin solution overnight and subsequently embedded in paraffin wax. Pancreatic tissue sections and various metastatic organs were cut at 5μm thickness and stained with hematoxylin and eosin. Immunohistochemical analysis was also performed on the same sections of pancreas isolated from the control and inhibitor treated mice for MUC4 antibody as described previously [42]. Immunoblotting analyses were also performed in tissue lysates for MUC4, pEGFR, EGFR, pHER2, HER2, phospho-STAT1, STAT1 and beta actin of control and canertinib treated mice as described previously [15].

### Statistical analysis

Student *t-*test was used to determine the statistical significance between control and treatment group in all the experiments pertains to this study. Statistical analysis and generation of graphs were performed using SigmaPlot (Version 11.2). P value less than 0.05 was considered to be statistically significant. Error bars were given on the basis of calculated S.E values.

## SUPPLEMENTARY MATERIAL FIGURES AND TABLE


