# Supplementary material for: Targeting EGF-receptor(s) - STAT1 axis attenuates tumor growth and metastasis through downregulation of MUC4 mucin in human pancreatic cancer
Source: Oncotarget. 2014 Dec 31;6(7):5164–81. doi: 10.18632/oncotarget.3286 (PMC4467140; doi:10.18632/oncotarget.3286)
Supplement: Supplementary file 1 [file oncotarget-06-5164-s001.pdf]

Targeting EGF-receptor(s) - STAT1 axis attenuates tumor growth and metastasis through downregulation of MUC4 mucin in human pancreatic cancer

Supplementary Material

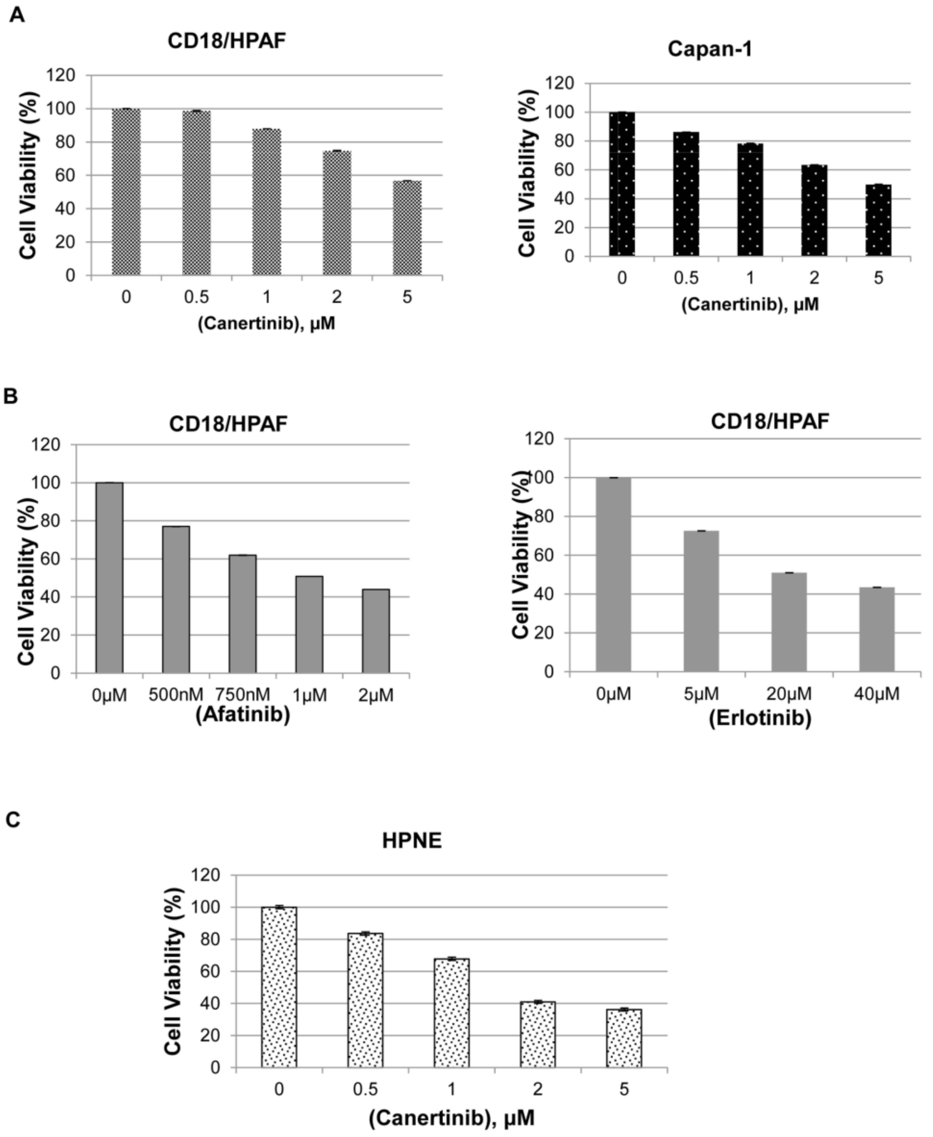

Figure S1: Canertinib, afatinib and erlotinib decreases cell viability in pancreatic cancer cells. **(A and B)** Pancreatic cancer cell viability was assessed with indicated concentration of canertinib, afatinib and erlotinib for 24h using the MTT assay. More specifically, canertinib was tested at different concentrations such as 0, 0.5, 1, 2, and 5µM,

afatinib at 0, 500 nM, 750 nM, 1μM, and 2μM and erlotinib at 0, 5, 20, and 40 μM. Our results demonstrate that treatment concentration of 0.5 μM concentration of canertinib had no effect on pancreatic cancer cell viability, whereas 1-5 μM concentration of canertinib led gradual decrease in cell viability in both CD18/HPAF and Capan-1 pancreatic cancer cells (98-56% and 86-49%, respectively). The effective concentration of 5 μM canertinib that inhibited cell viability by 50% was consider for further inhibitor (canertinib) studies in pancreatic cancer cells. Similarly, afatinib led to gradual decrease in cell viability of 77.02-43.93% and erlotinib inhibits proliferation within the range of 72.58-43.47% in CD18/HPAF cells. The effective 50% inhibitory concentration of 1 μM and 20 μM were considered for afatinib and erlotinib treatment respectively. **(c)** HPNE cells were treated with indicated concentration of canertinib and cell viability was assessed after 24 h.

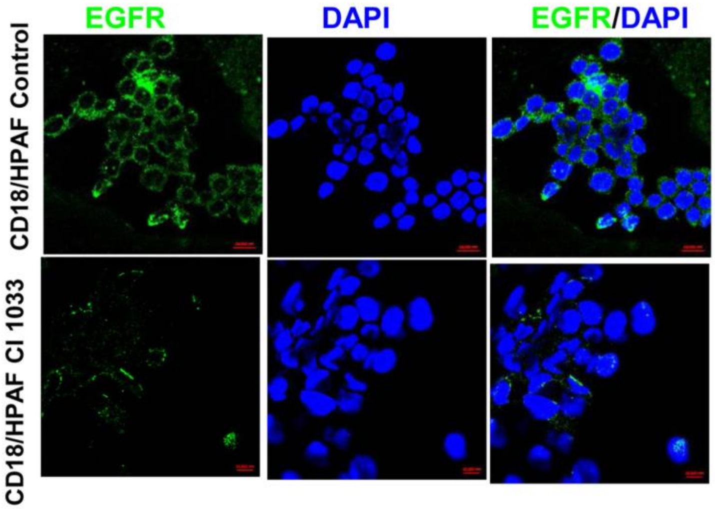

Figure S2: Canertinib down regulates membranous expression of EGFR in CD18/ HPAF pancreatic cancer cells. Confocal images represent the membranous down regulation of EGFR protein in CD18/HPAF cells on treatment with canertinib. Cells were counterstained with DAPI for nuclear staining.

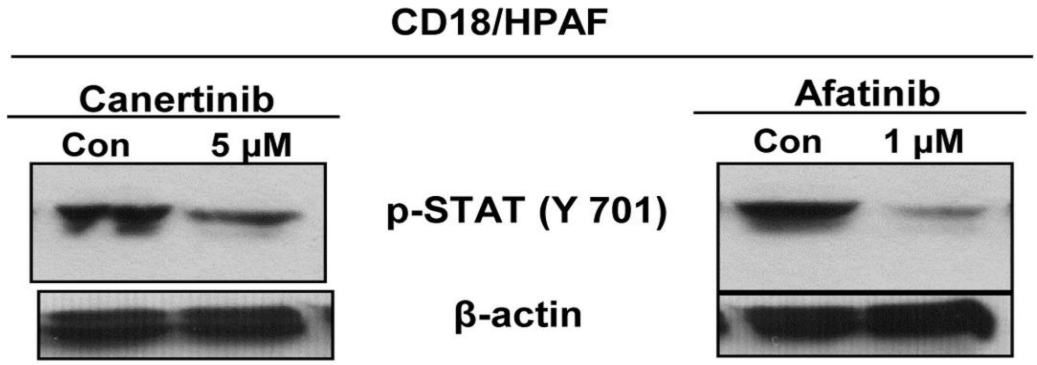

Figure S3: phosphorylation of STAT1 at tyrosine 701 was also decreased upon pan-EGFR inhibitors treatment. CD18/HPAF cells were treated with canertinib and afatinib at the indicated doses and analyzed for tyrosine phosphorylation of STAT1 at 701 by western blotting analysis.

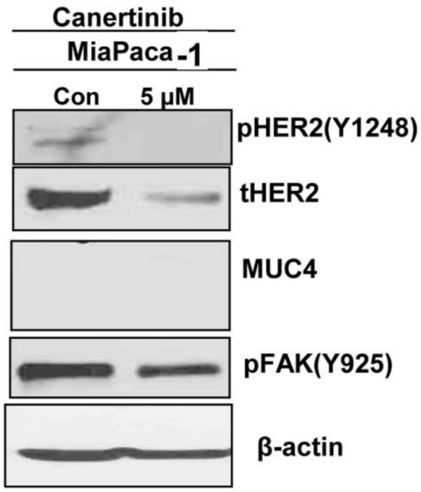

Figure S4: Canertinib is less sensitive in affecting pFAK in MUC4 negative pancreatic cancer cells. Endogenously MUC4 negative pancreatic cancer cell line, MiaPaca-1 was treated with canertinib at 5 μM concentration and assessed for proteins such as pHER2, total HER2, MUC4 and pFAK (Y925). Beta actin was used as loading control.

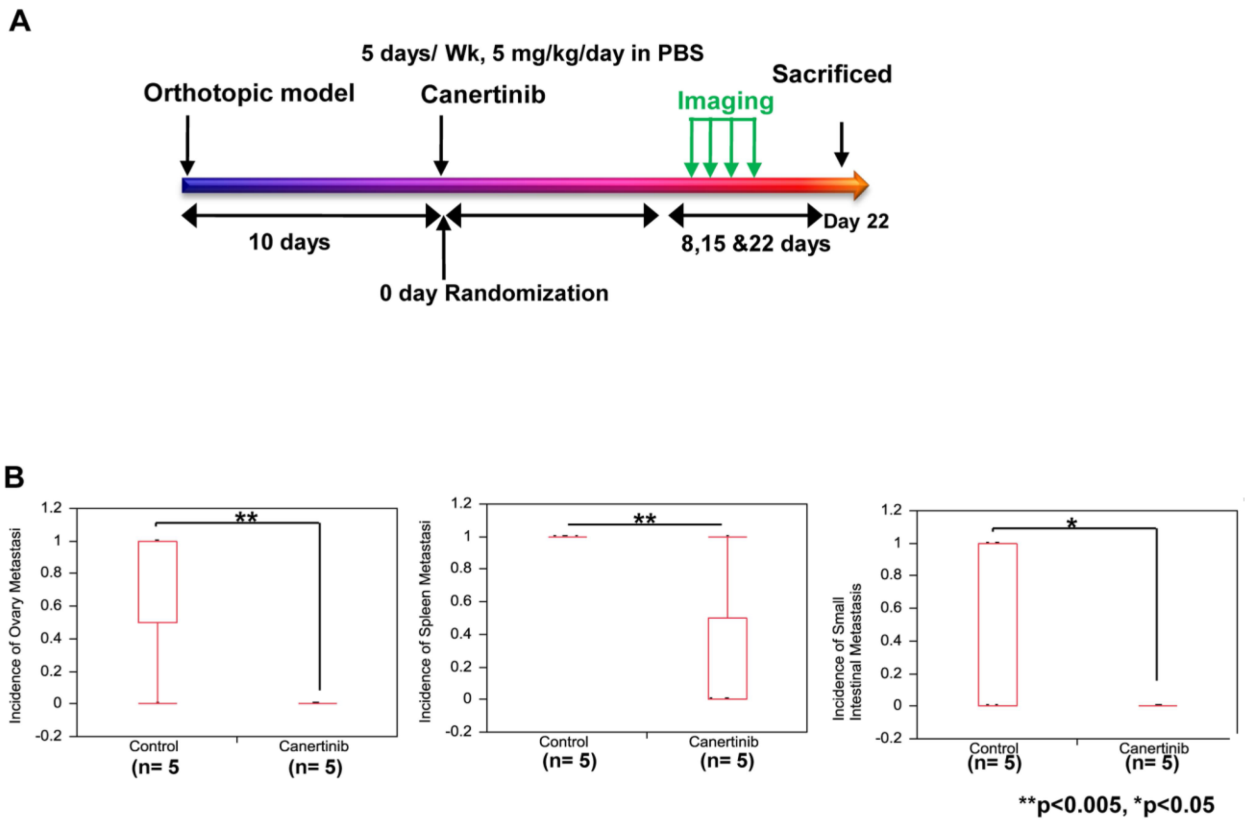

Figure S5: Pan-EGFR inhibitor treatment results in significant reduction of tumor growth and decreased incidence of metastasis. (A). Mice orthotopically implanted with CD18/HPAF luciferase tagged cells were treated with canertinib and a detailed description of experimental design, treatment strategies, drug dosage and detection system of pancreatic cancer orthotopic mouse model. (B). Canertinib inhibits metastasis of pancreatic cancer cells in various organs (*in vivo)*. Box plot shows a decrease in the incidence of metastasis to the other organs such as ovary ($p<0.005$), spleen ($p<0.005$) and small intestine ($p<0.05$) in the canertinib treated mice as compared to PBS treated control mice.

Supplementary table 1: Primary antibodies used in the study.

Antibody	Company name, Clone # and/or catalog#	Dilution	Analysis performed
Mouse monoclonal anti-MUC4	In house generated, 8G7 [9, 12, 13, 33].	1:1000<br>1:200	IB<br>IF
Rabbit monoclonal anti-EGFR	Cell Signaling Technology, ##4267, D38B1.	1:1000,<br>1:50	IB<br>IF
Mouse monoclonal anti-EGFR	In house generated (1C1) and previously described [33].	1:1000,<br>1:100	IB<br>IF
Rabbit polyclonal anti-HER2	Santa Cruz Biotechnology, SC-134481, H-200.	1:1000<br>1:100	IB<br>IF
Rabbit polyclonal anti-HER3	Santa Cruz Biotechnology, SC-285, C-17.	1:1000	IB
Mouse monoclonal anti-HER4	Santa Cruz Biotechnology, SC-8050, C-7.	1:500	IB
Rabbit polyclonal anti-STAT1	Cell Signaling Technology, #9172.	1:1000	IB
Rabbit monoclonal pan AKT (Total)	Cell Signaling Technology, #4691, C67E7.	1:1000	IB
Mouse monoclonal anti-ERK1/2	Santa Cruz Biotechnology, SC-135900, MK-1.	1:500	IB
Mouse monoclonal anti-FAK	Santa Cruz Biotechnology, SC-271195, B-8.	1:1000	IB
Rabbit polyclonal anti-Cyclin D1	Santa Cruz Biotechnology, SC-753, H-295.	1:1000	IB
Rabbit polyclonal anti-Cyclin A	Santa Cruz Biotechnology, SC-751, H-432.	1:500	IB
Rabbit monoclonal anti-phosphoEGFR (Y1068)	Cell Signaling Technology, #3777, D7A5.	1:1000	IB
Rabbit polyclonal anti-phosphoHER2 (Y1248)	Cell Signaling Technology,#2247S.	1:1000	IB
Rabbit monoclonal anti-phosphoHER3 (Y1289)	Cell Signaling Technology,#4791.	1:1000	IB
Rabbit monoclonal anti-phosphoAKT (Ser473)	Cell Signaling Technology, #4060X, D9E.	1:1000	IB
Rabbit polyclonal anti-phospho ERK1/2 (T202/Y204)	Cell Signaling Technology, #9101.	1:1000	IB
Rabbit polyclonal anti-phosphoFAK (Y925)	Cell Signaling Technology, #3284S.	1:1000	IB
Rabbit monoclonal anti-phospho STAT1 (Ser727)	Cell Signaling Technology, #8826, D3B7.	1:1000	IB
Rabbit monoclonal anti-phospho STAT1 (Y701)	Cell Signaling Technology, #9167S, 58D6.	1:100	IB
Mouse monoclonal anti-β-actin	Sigma-Aldrich, A1978, AC-15.	1:500	IB

Supplementary table 2: Pancreas weight of animal groups (weight in grams)

S.No	Animal groups		P value
	Control	Canertinib treatment	
1	889	578	0.016
2	1015	526	
3	730	596	
4	1887	1.008	
5	1898	535	
